# Adipose-derived cells improve left ventricular diastolic function and increase microvascular perfusion in advanced age

**DOI:** 10.1371/journal.pone.0202934

**Published:** 2018-08-24

**Authors:** Natia Q. Kelm, Jason E. Beare, Fangping Yuan, Monika George, Charles M. Shofner, Bradley B. Keller, James B. Hoying, Amanda J. LeBlanc

**Affiliations:** 1 Cardiovascular Innovation Institute, University of Louisville, Louisville, Kentucky, United States of America; 2 Kentucky Spinal Cord Injury Research Center, University of Louisville, Louisville, KY, United States of America; 3 Department of Physiology, University of Louisville, Louisville, Kentucky, United States of America; 4 Department of Pediatrics, University of Louisville, Louisville, Kentucky, United States of America; East Tennessee State University, UNITED STATES

## Abstract

An early manifestation of coronary artery disease in advanced age is the development of microvascular dysfunction leading to deficits in diastolic function. Our lab has previously shown that epicardial treatment with adipose-derived stromal vascular fraction (SVF) preserves microvascular function following coronary ischemia in a young rodent model. Follow-up studies showed intravenous (i.v.) delivery of SVF allows the cells to migrate to the walls of small vessels and reset vasomotor tone. Therefore we tested the *hypothesis that the i*.*v*. *cell injection of SVF would reverse the coronary microvascular dysfunction associated with aging in a rodent model*. Fischer 344 rats were divided into 4 groups: young control (YC), old control (OC), old + rat aortic endothelial cells (O+EC) and old + GFP^+^ SVF cells (O+SVF). After four weeks, cardiac function and coronary flow reserve (CFR) were measured via echocardiography, and hearts were explanted either for histology or isolation of coronary arterioles for vessel reactivity studies. In a subgroup of animals, microspheres were injected during resting and dobutamine-stimulated conditions to measure coronary blood flow. GFP^+^ SVF cells engrafted and persisted in the myocardium and coronary vasculature four weeks following i.v. injection. Echocardiography showed age-related diastolic dysfunction without accompanying systolic dysfunction; diastolic function was improved in old rats after SVF treatment. Ultrasound and microsphere data both showed increased stimulated coronary blood flow in O+SVF rats compared to OC and O+EC, while isolated vessel reactivity was mostly unchanged. I.v.-injected SVF cells were capable of incorporating into the vasculature of the aging heart and are shown in this study to improve CFR and diastolic function in a model of advanced age. Importantly, SVF injection did not lead to arrhythmias or increased mortality in aged rats. SVF cells provide an autologous cell therapy option for treatment of microvascular and cardiac dysfunction in aged populations.

## Introduction

While coronary artery disease (CAD) occurs in both older men and women, coronary microvascular dysfunction (CMD) is more often present in older women, who often exhibit signs and symptoms of CAD in the absence of obstructive coronary arteries [1]. At the same time, the incidence of left ventricular diastolic dysfunction (LVDD) increases in women after menopause and leads to heart failure [2]. One of the early manifestations of CAD is the development of microvascular dysfunction, and this can lead to changes in LVDD [3]. If left untreated, CMD leads to cardiomyocyte injury and myocardial stiffness and plays an important role in the pathophysiology of LVDD [4, 5].

While there are many treatment options available for CAD, including coronary artery bypass graft surgery, percutaneous transmural coronary angioplasty, and thrombolytic/pharmacological therapy [6], these are either impractical or have been relatively ineffective in persistently improving coronary microvascular function in an old female population [7–9]. Alternatively, cell therapy (using sources such as bone marrow and cardiac tissue) has been utilized in clinical trials to treat CAD and heart failure with moderate success, and this may be a more realistic treatment option for CMD. Relevant to this study, adipose tissue contains an easily isolatable, regenerative, and multipotent cell population defined as the stromal vascular fraction (SVF), and consists of endothelial cells, smooth muscle cells, blood cells, and mesenchymal cells containing perivascular and adventitial cells [10, 11]. SVF from adipose tissue has anti-apoptotic, anti-oxidant, anti-inflammatory, immune-modulatory effects via direct immunosuppressive/tolerance-inducing properties [12]. These positive characteristics of SVF has significantly raised the number of ongoing clinical trials using adipose-derived cells as a cell therapy [13].

Previous studies from our laboratory have shown enhanced coronary flow reserve (CFR, a measure of coronary microvascular function) following epicardial administration of SVF in a young rodent model of myocardial infarction, and this effect was independent of vascular density [14, 15]. CFR represents the myocardial blood flow ratio of hyperemic flow to rest and can be an indicator of CMD because it is a functional measure of large- and small-vessel ischemia [16, 17]. The aged rat model used in the present study exhibits similar age-related CMD that aged humans (especially female) demonstrate clinically [18–21], making them an ideal model to target therapeutically with our cell therapy. Recent collaborations with Morris *et al*. demonstrated that intravenously (i.v.)-delivered SVF was related to improved peripheral small artery function in a mouse model and revealed fluorescently-labeled SVF had migrated to the walls of small vessels and reset vasomotor tone [22]. *Therefore*, *we hypothesized that i*.*v*. *delivery of SVF cells can reverse age-related CMD and LVDD by incorporating into vascular walls and improving microvascular reactivity*. This study shows that old rats treated with SVF cells exhibited improved CFR, regional coronary perfusion, and LVDD compared to old control and old rats treated with a control cell population. We also identified incorporated SVF cells in the aged cardiac tissue four weeks following injection. However, we did not see the expected improvements in isolated coronary vasoreactivity post-SVF treatment in old rats.

## Materials and methods

### Animal model, groups, endpoint procedures

All animal surgeries were performed in accordance with protocols approved by the University of Louisville Institutional Animal Care and Use Committee (IACUC-approved protocol #16705) and the NIH *Guide for the Care and Use of Laboratory Animals* (9th ed., 2011).

The female Fischer-344 rat model was selected due to the inbred background of the animals, the ability to inject syngeneic cells with minimal immuno-rejection, the absence of large-vessel CVD as the colony ages, and the development of aging-induced CMD, which resembles the clinical scenario in aging humans [21]. Young (3 mo) and old (22 mo) female Fischer-344 rats (Harlan Laboratories, Indianapolis, IN, USA and National Institute on Aging, Bethesda, MA, USA, respectively) were housed in groups with free access to food and water and were maintained on a regular 12-hour light/dark cycle. Young rats were acclimated to facility conditions for a minimum of one week prior to endpoint procedures. Old rats were acclimated to facility conditions for a minimum of one week prior to “baseline” ultrasound scanning then divided into 3 groups, including old control (OC) and 2 cell injection groups: old + rat aortic endothelial cells (O+EC) and old + GFP^+^ SVF cells (O+SVF). After four weeks, old rats were 23–24 months at the time of endpoint procedures. All groups were randomly divided into subgroups for endpoint procedures: echocardiography, microspheres, isolated coronary arteriole experiments, or histology. Animals were deeply anesthetized with 5% isoflurane-balanced O_2_ before being euthanized by removal of the heart.

### Rat aortic endothelial cell isolation

Unlabeled rat aortic endothelial cells (RAEC) were obtained at passage 3 from Angio-Proteomie (Boston, MA). RAEC were grown in RAEC culture media (RCM: DMEM, FBS, HEPES, L-glutamine, ECGS) on 1% gelatin coated flasks in 5% CO2 incubator. Media was changed every other day. Cells were passaged upon reaching ~80% confluency and split 1:3 until trypsinized at passage 5–6.

### SVF isolation

A GFP^+^ Fischer-344 rat colony (aged to 3–6 months in house) was used as SVF donors. Briefly, the fat pad of the uterus was dissected from fully anesthetized donors and put into a 50 mL conical tube containing 0.1% BSA-DCF/PBS. Care was taken to avoid the large vascular plexus of the uterus during dissection. 15–20 mL of adipose tissue was collected for each isolation. Fat tissues were minced for 10 minutes with scissors, then incubated with digestion solution containing 0.75x collagenase DE40 (VitaCyte, 011–1130); and 1x of DNase (Sigma, DN25-1G) in 1.5x of 0.1% BSA-DCF/PBS to total volume of the fat for 35 minutes at 37 ^o^C with rotating agitation. The cell-digestion mixture was centrifuged at 400 g for 4 minutes to get gradient layers. After removing top layers of adipocytes and supernatant, the top portion of the cell pellet was collected and added to 0.1% BSA-DCF/PBS to disperse the cells; the lower red blood cell layer was discarded [15]. Briefly, this cell population has been shown to exhibit ~12±5% CD34 (hematopoietic and endothelial stem cell marker), 19±3% VEGFR2 (VEGF receptor type 2, Flk-1), 9±3% cKit (tyrosine kinase receptor that binds to stem cell factor), 10±4% CD31 (endothelial marker), and 32±9% CD11b (monocyte/macrophage marker) [23].

### Injection protocol

The cell solutions (RAEC or SVF) were filtered through a 20um screen to eliminate large cell and tissue aggregates. Cell count was determined using a NucleoCounter® SP-100™. GFP^+^ was confirmed via fluorescence microscopy prior to injection of the SVF solution. Old rats were intravenously injected with 6x10^6^ of either RAEC or GFP^+^ SVF cells in 1ml lactated-ringers solution (warmed to 37°) via the tail vein into 22-month-old rats. Cell concentration was chosen to remain consistent with ongoing studies in the laboratory.

### Measurement of cardiac function

#### a. Echocardiography

Echocardiography was performed with Vevo 3100, using 250 MHz linear probe (FUJIFILM VisualSonics Inc., Toronto, Ontario, Canada). Rats were anesthetized with isoflurane and maintained at a surgical depth of anesthetic (induction chamber at 5% with 1.5–2.0 l/min O2 flow followed by 1.5–2.0% with 1.5–2.0 l/min O2 flow) and placed in a supine position, and their thorax was shaved. Body temperature was maintained at 37–38°C, and heart rate was monitored using Vevo Imaging Station. The E/A ratio was obtained from an apical four chamber view with conventional pulsed wave Doppler. E/A ratio was calculated from the peak velocity flow in early diastole (the E wave) to peak velocity flow in late diastole caused by atrial contraction (the A wave) during resting conditions. After the acquisition of images during rest, the tail was cleaned and the tail vein cannulated with a 25-gauge butterfly needle. Hyperemic blood flow (BF) “stress” was accomplished through i.v. infused dobutamine (Hospira, Lake Forest, IL, USA) in a stepwise manner with progressively increasing doses (5, 10, and 20 g·kg^-1^ ·min^-1^, 4’/stage) using an automated perfusion pump to reach a peak response (a plateau in the HR) [24, 25]. An image was captured along the parasternal short axis during the last 30 s of each dose and analyzed offline using the VEVO^®^ LAB software. Standard measures of LV structure [i.e., LV internal diameter (LVID) and LV posterior wall thickness (PWT)] and function [i.e., stroke volume (SV), cardiac output (CO), and ejection fraction (EF)] were obtained along the parasternal short axis during resting and stress conditions. In M-Mode, wall thicknesses and chamber dimensions across the sample line are used to calculate anatomical and functional parameters [26]. End-diastolic and end-systolic volume were estimated from LVID at systole. Results from five cardiac cycles during expiration were averaged together and used for between-group and within-group (time after 4 weeks of treatment) comparisons. Number of animals in each group: YC n = 10, OC n = 9, O+EC n = 10, O+SVF n = 9

#### b. Coronary flow reserve

In addition to the standard echocardiographic imaging of cardiac function, a modified parasternal short-axis projection was used for Doppler recording of the blood flow velocity of the left anterior descending artery (LAD) at rest while the animals were anesthetized with 1.5% inhaled isoflurane, and again during the last 30 seconds of each dobutamine dose described above [26]. Dobutamine is one of the most potent inotropes and is a cardioselective β1-adrenoreceptor agonist that increases CO and myocardial BF and contractility. LAD velocity pre-dobutamine and during the dobutamine stress challenge were averaged from three consecutive cardiac cycles and CFR was calculated as the ratio of the mean peak LAD velocity values during stress and rest [27].

#### c. Mapping functional blood flow perfusion

Prior to explant, microspheres were delivered via apical injection as previously described in order to evaluate potential regional blood flow changes in a subset of animals from each group [14]. Briefly, 1.2x10^6^ nonradioactive elementally-labeled 15-um neutron-activated microspheres (Biopal) were injected into the LV transapically while simultaneously sampling carotid blood at a known withdrawal rate. Two separate isotopes were injected: one at rest and one after a 5-minute dobutamine injection (10 ug/kg/min) [28]. Afterward, the LV is sectioned as described previously [14]. Percent above resting BF was calculated as [(dobutamine BF–resting BF)/(resting BF) *100]. Number of animals in each group: YC n = 11, OC n = 11, O+EC n = 4, O+SVF n = 5.

### Subepicardial arteriole isolation experiments

The heart was removed from a subset of animals in each group. Coronary arterioles from the LAD artery distribution were isolated and transferred to a vessel chamber, cannulated on both ends, then pressurized to 45 mmHg [29]. Vessel diameter was manually measured with a video caliper (Colorado Video, Boulder, CO). Vessels without leaks were allowed to develop spontaneous tone (≥ 20% constriction from initial diameter).

The order of the following experimental periods was randomized in each vessel to ensure that responses were neither interactive nor time-dependent. Myogenic response was evaluated by recording diameter changes in response to graded increases in pressure, 0–90 mm Hg at 15 mm Hg increments (3’ stages). Concentration-response curves to bradykinin (3’ stages, 1e^-13^ - 1e^-7^ M), an endothelium-dependent vasodilator, and endothelin (3’ stages, 1e^-11^ - 3e^-8^ M), a potent coronary vasoconstrictor, were performed. At the conclusion of each experiment, the vessels were washed 2x20 min with Ca^2+^-free PSS. Maximum diameter (Dmax) was determined as the largest diameter achieved throughout the experiment. Normalized diameter = Ds/Dmax, where Ds is the steady state diameter measured after each pressure stage. Number of animals in each group: Endothelin response: YC n = 9, OC n = 24, O+EC n = 9, O+SVF n = 15; Bradykinin response: YC n = 8, OC n = 18, O+EC n = 6, O+SVF n = 14; Myogenic response: YC n = 12, OC n = 23, O+EC n = 8, O+SVF n = 17.

### Mesenteric arteriole isolation experiments

To assess potential peripheral vascular effects from cell treatment, the mesenteric artery was chosen. Following explant of the heart in all groups, the vascular tree from each animal was cut from the first-order branch point to the end of the third-order branch of the superior mesenteric artery. In a cold dissection bath, the superior mesenteric artery and its branches were dissected from the section of the mesenteric vascular bed that feeds the jejunum 3–12 cm distal to the pylorus. Third-order vessels were isolated and transferred to a vessel chamber, cannulated on both ends, then pressurized to 50 mm Hg [30]. Vessel diameter was measured with a video caliper (Colorado Video, Boulder, CO). Vessels without leaks were allowed to warm up to start an experiment and phenylephrine (2uM) was added to obtain pre-constriction [31].

The order of the following experimental periods was randomized in each vessel to ensure that responses were neither interactive nor time-dependent. Endothelium-dependent dilation was pharmacologically evaluated by ACh (1x10^-9^-1x10^-4^M), and repeated in the presence of L-NMMA (10 μM) to assess NO-mediated vasodilatory contribution. Endothelium-independent dilation in response to sodium nitroprusside (SNP, 1x10^-9^-1x10^-4^M) was determined. At the conclusion of each experiment, the vessels were washed with Ca^2+^-free PSS containing 100um SNP to determine Dmax. Number of animals in each group: ACh, n = 9 for OC, n = 5 for Old+EC, n = 7 for Old+SVF; for SNP, n = 7 for OC, n = 5 for Old+EC, n = 7 for Old+SVF.

### Histology

A subset of hearts from each group were embedded in OCT blocks immediately after KCl injection (40mM). Eight-μm cryostat sections were cut for immunofluorescence (IF) and histology staining. Slides for histology were stained with haematoxylin and eosin (H&E) for LV thickness measurement; adjacent sections were stained with Masson’s trichrome stain to assess collagen composition of the hearts. H&E-stained serial sections were examined for quantitative morphometry using a brightfield Nikon Eclipse E600 microscope with a 1x air objective at the papillary muscle level, one image per animal [32]. Wall thickness (mm) was determined by dividing the heart into 8 equal sections and averaging the length of the sections from chamber to epicardium. For collagen content analysis, three 8-μm sections from the middle of each heart to apex were selected for Trichrome staining, 50 μm between each section. Trichrome Stain (Masson) Kit (Sigma, HT15) was utilized; the blue color surrounding the vessels and in the interstitial area were defined as collagen. Images were acquired with Olympus U-TV0 63XB camera, 4x/0.13 UPlan FLN objective and Olympus cellSens software. At least three fields of each heart section were randomly acquired. Areas of collagen and total area of the images were calculated by color threshold setting in ImageJ software, and the ratio of collagen to total area was calculated.

For IF staining procedure, after the frozen sections were fixed in 4% PFA then rinsed in 1xPBS, slides were blocked with 0.1 M Glycine/0.5% Triton X-100, followed by PBS wash. Sections were incubated with primary antibodies Goat anti-GFP (ab6673, 1:100, Abcam, Cambridge, UK); Mouse anti-ED1 (ab31630, 1:100, Abcam, Cambridge, UK); Rabbit anti-NG2 (ab6673, 1:100, Abcam, Cambridge, UK) and/or GS1 lectin (RL-1102, 1:200, Vector, Burlingame, USA) which were diluted in 1.5% BSA/PBS overnight at 4°C. On the second day, sections were washed with PBS and incubated with secondary antibodies (A11055, Alexa Fluor 488-conjugated Donkey anti-Goat; A21202, Alexa Fluor 488-conjugated Donkey anti-Mouse; A21203, Alexa Fluor 594-conjugated Donkey anti-Mouse; A31573, Alexa Fluor 647-conjugated Donkey anti-Rabbit, Molecular Probes) in 5% donkey serum/PBS at RT, followed by PBS wash. Slides were sealed with anti-fade mounting medium with DAPI for nuclei counter-stain (P36935, Molecular Probes).

Nikon ECLIPSE confocal microscope system and Nikon NIS Elements AR software (Nikon, Melville, NY, USA) with 405, 488, 562, and 641 nm laser lines was used to acquire images. Images for cell quantification analysis were captured at 1024x1024 pixel density and 1 μm z-step (between 7–9 stacks) with a 40x oil immersion objective. Five images of each section were acquired randomly.

Using ImageJ Plugins tool, total nuclei (DAPI) in each image were counted for total cell numbers, ED1, GS1, NG2, ED1/NG2, ED1/GS1, and GS1/NG2 positive cells in each of the four experimental groups (total 5 images counted and averaged from each heart: YC n = 4, OC n = 3, O+EC n = 3, O+SVF n = 7); GFP, GFP/ED1, GFP/NG2 and GFP/GS1 positive cells were also counted in SVF-injection samples. Ratio of positive cells of targeted marker to total cells was calculated. Number of animals in each group for trichrome data, YC: n = 3, OC n = 3, O+SVF n = 4.

### Telemetry

A small subgroup of OC (n = 4) and O+SVF animals (n = 5) received indwelling telemetry units to assess potential arrhythmias caused by SVF injection. Isoflurane anesthesia (induction chamber at 5% with 1.5–2.0 l/min O2 flow followed by 2.0–1.5% with 1.5–2.0 l/min O2 flow) was used for all surgical procedures. Briefly, a battery-operated transmitter (Data Sciences International, St. Paul, MN) was inserted into the abdominal cavity, which allows bipolar lead electrocardiogram (ECG) recordings corresponding to vectorcardiographic Y lead (craniocaudal direction). The transmitter body was placed in the abdominal cavity, one electrode (non-inverting) was fixed to the dorsal surface of the xyphoid process, and the other electrode (inverting) was placed in the anterior mediastinum close to the right atrium. Immediately after surgery, rats were individually housed and allowed 7 days of recovery before the start of ECG recordings. ECG signals were picked up by a radiotelemetry receiver (RPC-1) placed under the housing cage and recorded via Ponemah 5.2 data acquisition system (Data Sciences Int., St. Paul, MN, USA).

During the follow-up period, 30-minute continuous ECG recordings were performed in aging animals once a week for four weeks, on the same day of the week, between 9 and 11 am. ECG signals were analyzed off-line in order to measure: RR interval duration (the time interval between two consecutive R wave peaks), PQ interval (time between atrial and ventricular depolarization), QRS complex duration, QT interval (time between QRS complex and ventricular repolarization), QTc interval (heart rate-corrected QT interval, or Bazett's formula), and number of arrhythmic events.

### Statistical analyses

Statistical analyses were performed with SPSS Version 22 (IBM Corp, Armonk, NY, USA), and the significance level was set at p ≤ .05. All baseline cardiac data were analyzed using one-way ANOVA followed by Bonferroni *post hoc* test or repeated-measures (RM) ANOVA where appropriate. Concentration-response comparisons were analyzed with RM ANOVA. All significant main or interaction ANOVA effects were further investigated using Bonferroni highly significant difference *post hoc* testing. Data are represented as means ± SD unless indicated otherwise.

## Results

### Animal characteristics and echocardiographic parameters

**Fig 1:**
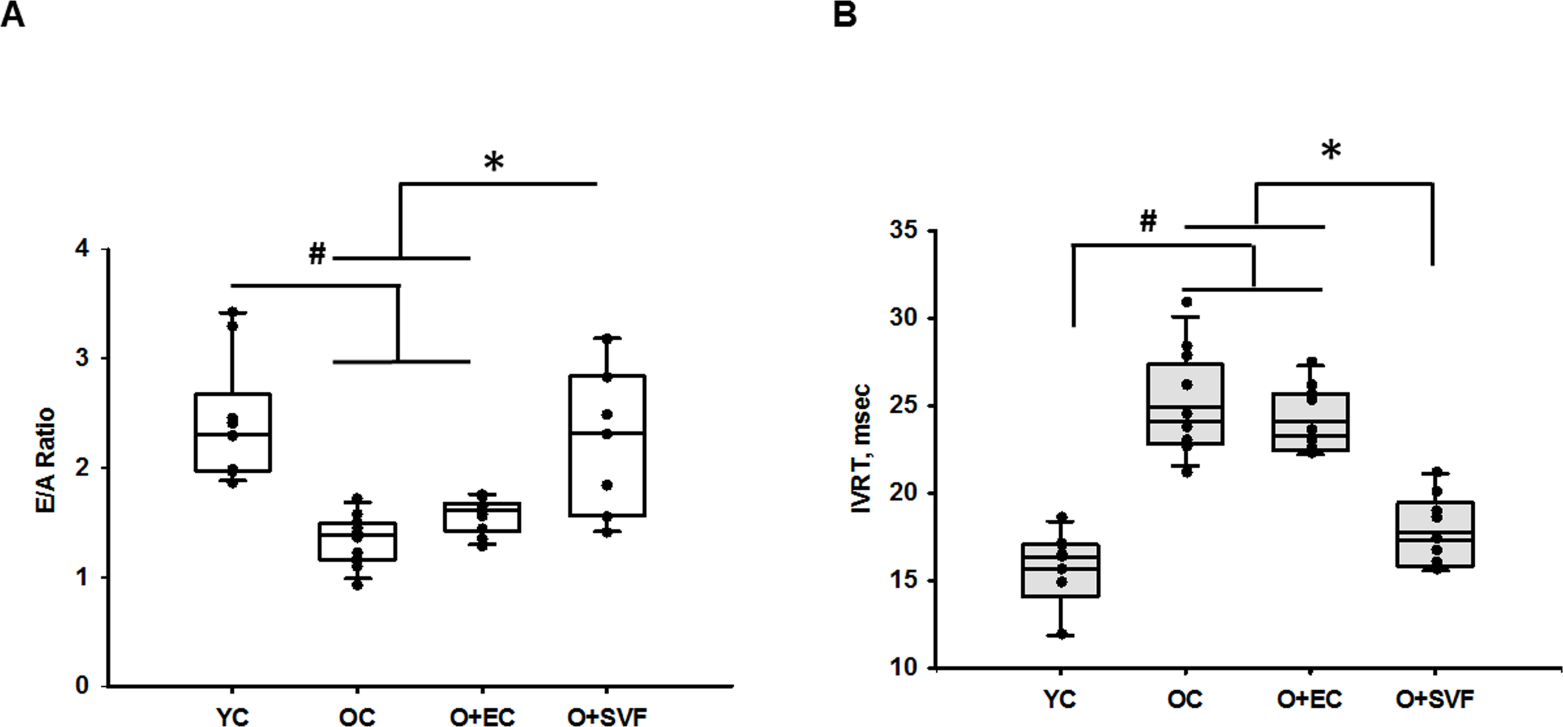
Diastolic function assessment using echocardiography. The graphical representation of E/A ratio shows significant reduction of diastolic function in OC animals, which was rescued by SVF treatment;(A). The graphical representation of IVRT shows a significant increase of IVRT in OC and O+EC animals, which was rescued by SVF treatment; (B) *P* ≤ 0.05 vs. Old+SVF (*) and vs. Young Control (#); Data are presented as mean ± SD, analyzed with one-way ANOVA followed by Bonferroni post hoc test of following number of animals in each group: YC n = 9, OC n = 12, O+EC n = 10, O+SVF n = 8.

To determine whether the systemic delivery of SVF or EC impacted basic animal characteristics and LV function (systolic and diastolic function), we collected gross anatomical and ultrasound cardiac measurements; these data are presented in **Table 1**. All old groups (OC, O+EC, and O+SVF) exhibited increased body weight (BW), total ventricular weight (HW), and LV weight compared to YC, but the ratio of HW/BW from old groups with or without treatment was not different from YC. No significant increase in mortality was reported in O+SVF group compared to OC, but we did observe one mortality case in O+EC group. Echocardiographic parameters measured at rest prior to explant are summarized in **Table 1**. All old rats demonstrated increased stroke volume due to higher end systolic volume and end diastolic volume regardless of treatment, but CO was similar to YC (**Table 1**). Fractional shortening in old rats was slightly lower but not significantly different from YC (**Table 1**) and stayed within normal limits for all groups. In order to assess diastolic function, E/A ratio was calculated in all groups at rest. E/A ratio was decreased in OC (1.38 ± 0.24, n = 12) compared to YC (2.41±0.58, n = 9, p<0.005) without accompanying changes in HR, CO, or systolic function assessed by ejection fraction (**Table 1, Fig 1**). E/A ratio was significantly increased in old rats following SVF treatment (2.29 ± 0.22, n = 8) compared to OC and O+EC (**Fig 1B**). We observed similar changes in isovolumic relaxation time (IVRT) obtained from ultrasound; IVRT was significantly increased in old rats with or without EC treatment (OC-23.99± 2.0, n = 12, O+EC-23.97± 1.86, n = 10), which was reduced after SVF treatment (SVF-18.00± 1.9, n = 8) back to YC levels (16.05± 1.88 n = 9).

**Table 1:** Animal characteristics and echocardiographic parameters. Echocardiographic values were averaged during resting conditions prior to dobutamine challenge. CFR (baseline) was assessed after initial acclimatization in old animals prior to assigning treatment.

	YC	OC	O + EC	O + SVF
age (months)	3.5±0.5	23±1*	23±1*	23±1*
Body Weight (g)	200±36	264±19*	249±24*	241±16*
Heart Weight (mg)	477±34	643±52*	650±45*	616±54*
HW/BW (mg/g)	2.45±0.2	2.47±0.2	2.638±0.1	2.6±0.3
LV Weight (mg)	384±47	526±43*	524±35*	506±38*
LV Length (mm)	0.939±0.036	1.62±0.0059*	1.54±0.00334*	1.367±0.0347*#
Heart Rate (bpm)	340±25	330±28	343±18	317±34
Volume; s (uL)	16±5	36±5*	30±12*	40±15*
Volume; d (uL)	136±21	184±32*	176±28*	188±25*
Stroke Volume (uL)	120±17	146±27*	146±27*	148±25*
IVS;d	1.38±0.05	1.69±0.06*	1.66±0.07*	1.46±0.11
IVS;s	2.58±0.13	2.83±0.12*	2.95±0.24*	2.54±0.25
LVPW;d	1.52±0.17	2.19±0.24*	2.01±0.11*	1.64±0.25
LVPW;s	2.50±0.2	3.12±0.24*	3.22±0.41*	2.67±0.24
SV/BW (uL/g)	0.64±0.119	0.56±0.120	0.62±0.120	0.63±0.112
Cardiac Output (mL/min)	40±5	48±8	50±10	46±7
Ejection Fraction (%)	87±2.9	80±2	83±5.8	81±4.5
CFR (baseline)	N/A	1.6±0.05*	1.4±0.07*	1.6±0.09*
CFR	2.24±0.06	1.7±0.05*	1.5±0.05*	2.3±0.2

*Statistically significant difference between mean values compared to YC group.

#Statistically significant difference between mean values compared to OC group.

Data are presented as mean ± SEM, analyzed with one-way ANOVA followed by Bonferroni post hoc test of following number of animals in each group: YC n = 10, OC n = 9, O+EC n = 10, O+SVF n = 9

**Fig 2:**
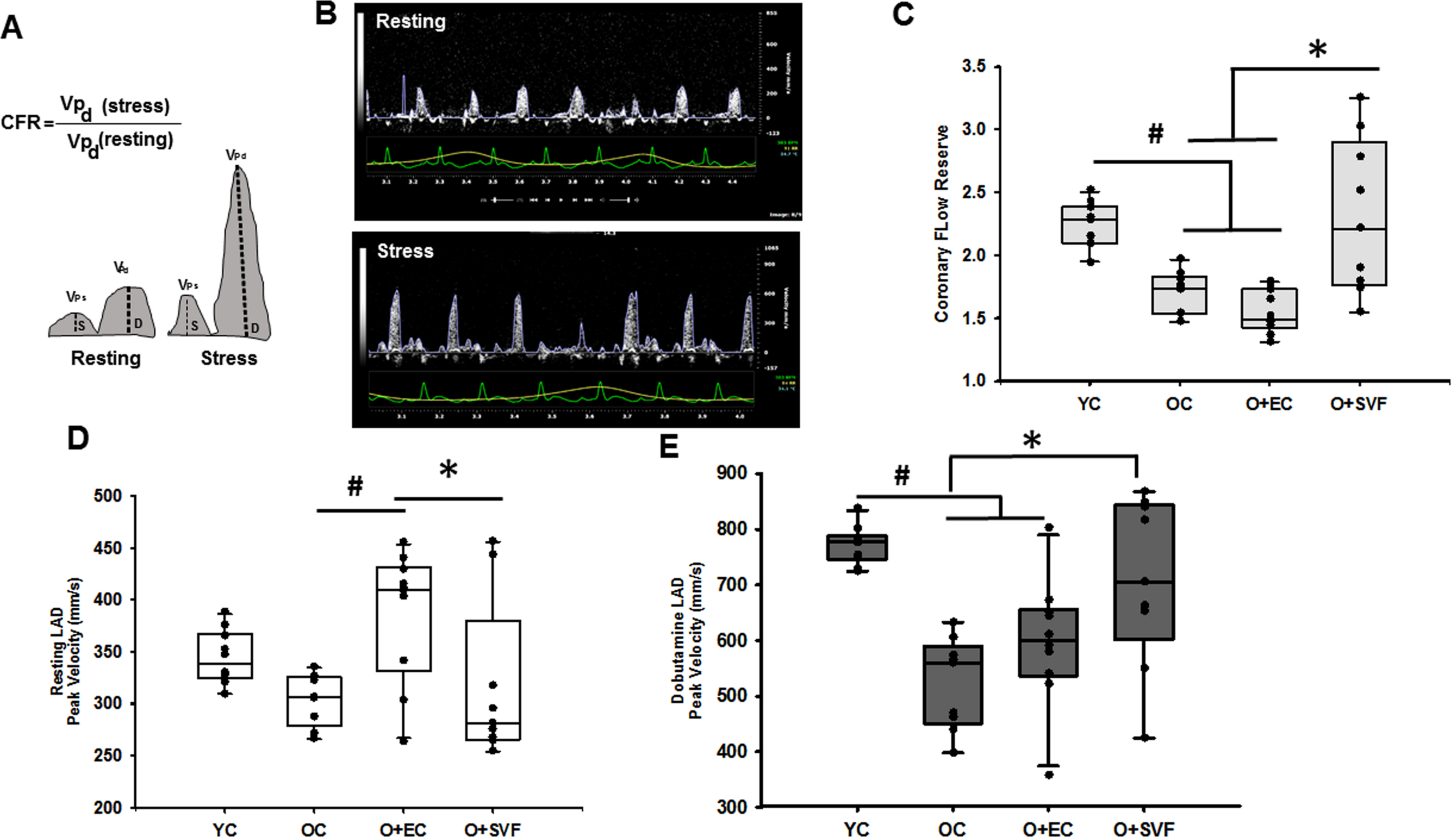
Measurement of coronary flow using Doppler echocardiography in rats. Graphical representation of CFR calculation (A), Doppler velocity waveforms from the LAD were traced and peak velocity was measured during rest (scale bar = 850 mm/s) and during stress (Dobutamine infusion, scale bar = 1065 mm/s) (B). CFR was calculated from the peak velocities in our experimental groups (C). LAD peak velocity during rest (D) and Dobutamine-induced stress (E). *P* ≤ 0.05 vs. Old+SVF (*) and vs. Young Control (#); Data are presented as mean ± SD, analyzed with one-way ANOVA followed by Bonferroni post hoc test of following number of animals in each group: YC n = 10, OC n = 9, O+EC n = 10, O+SVF n = 9.

In order to evaluate resting heart function and the response to a stimulus, Doppler recording of the blood flow velocity of the LAD was obtained in resting and dobutamine conditions (**Fig 2A and 2B)**. Absolute resting LAD flow velocities showed a slight increase in O+EC, but there were no differences between young and old groups with or without SVF treatment (**Fig 2D**). At the end of dobutamine infusion, the YC group increased absolute LAD velocity (771.95 ± 34.15mm/s, n = 10) (**Fig 2E**) but this response was diminished in OC group (523.11 ± 81.98 mm/s, n = 9) and O+EC (597.30 ± 115.29 mm/s, n = 10). Post-dobutamine LAD velocity was significantly improved after treatment with SVF in old animals (707.89 ± 152.03 mm/s, n = 9) compared to OC but not O+EC (**Fig 2E**). CFR was assessed because it is a functional measure of large- and small-vessel ischemia and is an indicator of coronary microvascular function. CFR was significantly reduced in all old groups prior to treatment at the baseline scan compared to the YC group (**Table 1**). Four weeks following SVF injection, CFR was significantly increased (2.31 ± 0.61, n = 9) compared to OC (1.71 ± 0.17, n = 9) and O+EC (1.67 ± 0.20, n = 10), resulting in no statistical difference between O+SVF and YC (2.25 ± 0.18, n = 10, p<0.005) (**Fig 2C**).

### Quantitative analysis of heart perfusion with microspheres

**Fig 3:**
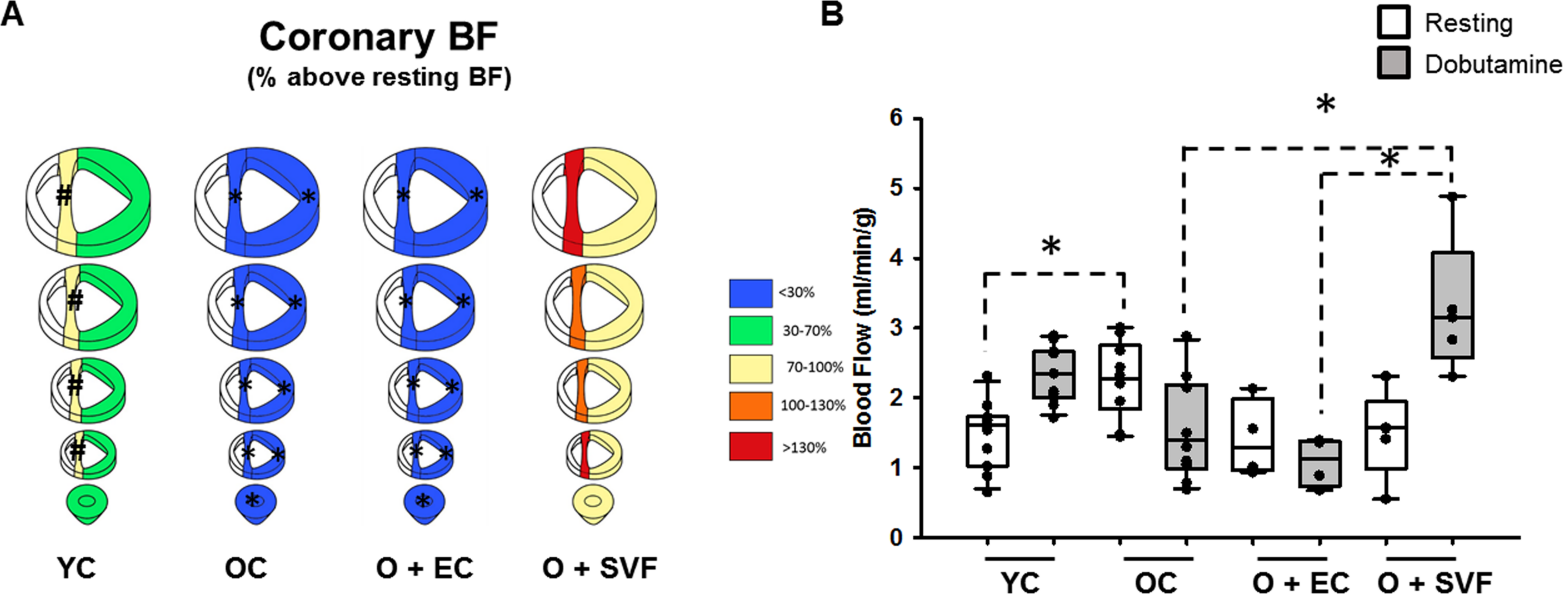
Measurement of heart perfusion using microspheres. Coronary blood flow was evaluated by the injection of two different stable isotope-labeled microspheres during rest and dobutamine infusion. After explant, the LV was divided into 4 rings above the apex up to papillary region, then rings were separated by septum and free LV wall. Percent blood flow above resting for each section is depicted on the left, *P* ≤ 0.05 vs. O+SVF (*) and vs. OC (#) (A). Absolute LV blood flow (ml/min^-1^.g^-1^, averaged across all sections of one heart) during rest was significantly higher in OC compared to YC, p < .05 (#); following dobutamine challenge, O+SVF showed increased blood flow compared to OC and O+EC, p < .05 (*) (B). Data are presented as mean ± SD, analyzed with one-way ANOVA followed by Bonferroni post hoc test of the following number of animals in each group: YC n = 11, OC n = 11, O+EC n = 4, O+SVF n = 5.

In order to evaluate regional coronary microvascular perfusion in our groups, isotope-labeled microspheres were injected into the apical region under resting and dobutamine-stimulated (stress) conditions prior to explant. A significant age-related decline in BF % was seen in the OC group (**Fig 3A**, YC: 30–70% vs OC: <30%). This deficit was eliminated in old rats following SVF treatment (**Fig 3A**, O+SVF: >70%). The resting LV blood flow was significantly higher in OC compared to YC, but did not increase following dobutamine infusion (**Fig 3B**). Dobutamine-stimulated LV flow was enhanced in old rats treated with SVF compared to OC and O+EC, indicating enhanced responsiveness under stress conditions (**Fig 3B**).

### Isolated coronary arteriole responses: Endothelin, bradykinin, and myogenic tone

**Fig 4:**
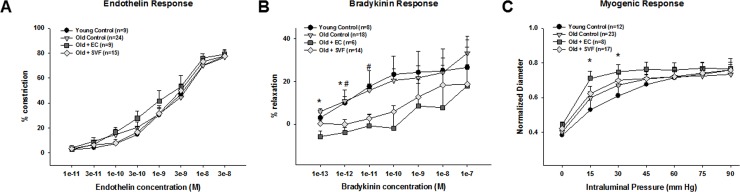
Isolated coronary arteriole vasoreactivity. Endothelin-induced vasoconstriction in coronary arterioles was similar between all groups (A). Coronary arterioles from O+EC or O+SVF decreased sensitivity to the low doses of bradykinin (O+EC group in 1e^-13^ and 1e^-12^ concentrations and O+SVF in 1e^-12^ and 1e^-11^ concentrations). Main group effect, *P* < 0.001, *P* ≤ 0.05 (YC and OC) vs. O+EC (*) vs O+SVF (#) (B). Myogenic response was higher in O+EC group during the 15 and 30mm Hg intramural pressure. Main group effect, *P* < 0.001, *P* ≤ 0.05 YC vs. O+EC (*) (C). Data are presented as mean ± SEM, analyzed with repeated measures ANOVA followed by Bonferroni post hoc test of the following number of animals in each group: Endothelin response: YC n = 9, OC n = 24, O+EC n = 9, O+SVF n = 15; Bradykinin response: YC n = 8, OC n = 18, O+EC n = 6, O+SVF n = 14; Myogenic response: YC n = 12, OC n = 23, O+EC n = 8, O+SVF n = 17.

To determine if SVF treatment altered coronary vasoreactivity, coronary arterioles were isolated and responses to pressure, vasodilation and vasoconstriction was assessed. Coronary arterioles (<200μm) were chosen because they represent the area of the microvasculature where alterations in vasoreactivity affect overall coronary perfusion the most [29]. Endothelin-induced vasoconstriction in coronary arterioles was similar between all groups (**Fig 4A**). Response to bradykinin was not different between YC and OC (**Fig 4B**), but coronary arterioles from O+EC or O+SVF exhibited decreased sensitivity to the low doses of bradykinin (O+EC group in 1e^-13^ and 1e^-12^ concentrations and O+SVF in 1e^-12^ and 1e^-11^ concentrations). Response was restored in higher concentrations of bradykinin in cell-injection groups, eliminating any differences between all groups (**Fig 4B**). Myogenic response was higher in O+EC group during the 15 and 30mm Hg intramural pressure (**Fig 4C**); but after 45mm Hg there was no significant difference between the groups (**Fig 4C**). The Dmax was significantly increased in OC vs YC (YC: 121.544±16.7um vs. OC: 150.25±25um) but there was no significant difference among old cell-injected groups (O+EC:143.4±21.6um, O+SVF: 133.84±31.61um).

### Isolated mesenteric arteriole responses: Acetylcholine, sodium nitroprusside

In order to determine if treatment with SVF altered vascular reactivity in non-cardiac tissue, mesenteric arteries from the periphery were assessed for endothelium-dependent and -independent vasodilation. Third-order mesenteric arteries were chosen because they represent the area of the intestinal vasculature where potential alterations in vasoreactivity affect overall blood flow changes the most [33]. Initial diameter after phenylephrine administration was similar between old groups (OC: 65.3 ± 9.8um, O+EC: 70.2 ± 17.39um, and O+SVF: 70.1 ± 16um). Surprisingly, mesenteric arteries from YC rats could not maintain phenylephrine pre-constriction in order to test endothelial dependent/independent vasodilatation. Endothelial-dependent and–independent dilation were not altered in old rats regardless of treatment (**S1 Fig**). Dmax was not significantly different among old experimental groups (OC: 219.6 ± 14.21um, O+EC: 226 ± 13.99um, and O+SVF: 218 ± 15.68um).

### Histology

**Fig 5:**
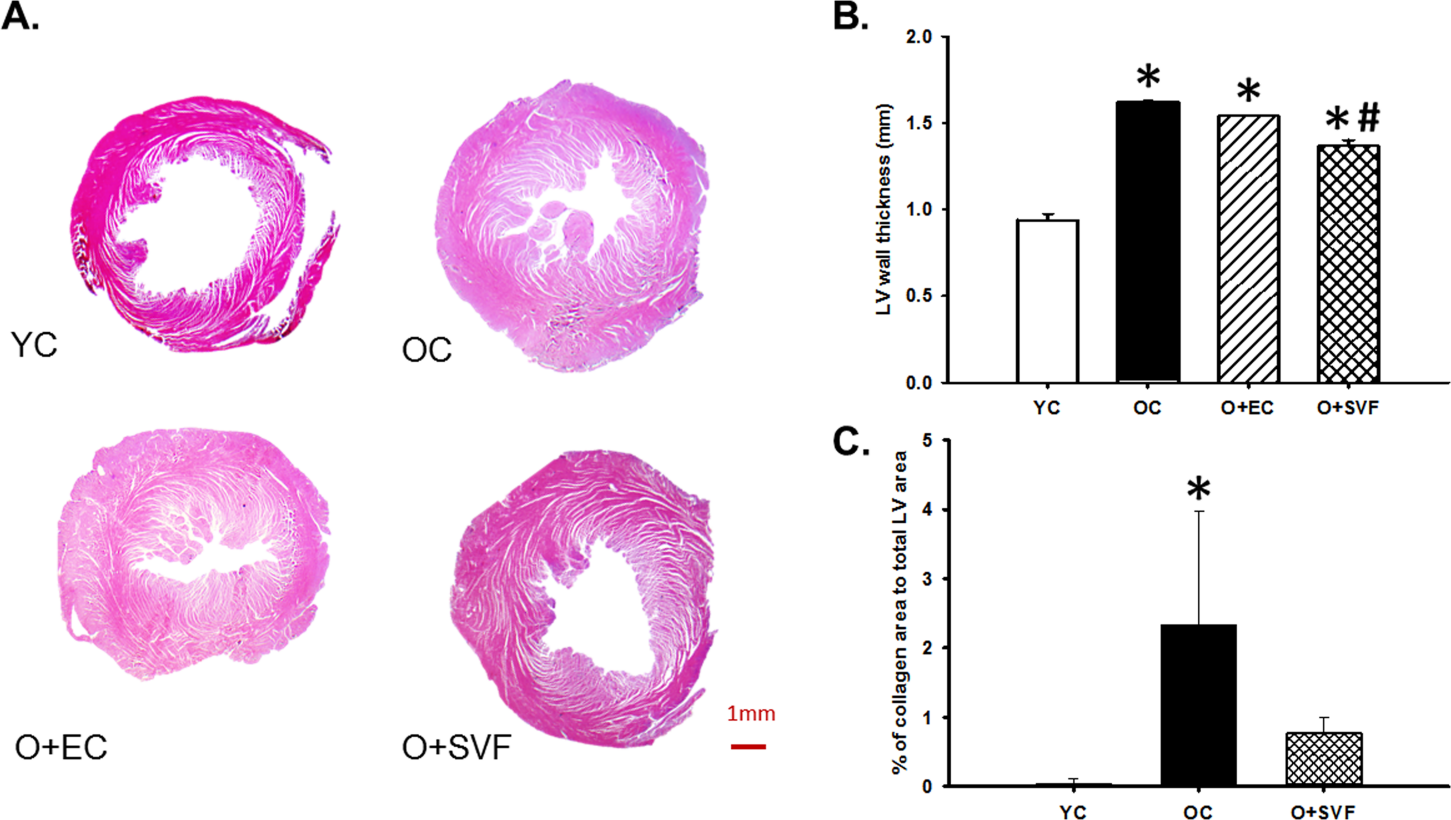
Representative H&E staining of heart sections and trichrome analysis. Examples of cross-sectional view of the whole hearts at the papillary level from the experimental groups, scale bar = 1mm (A). All old groups exhibited increased wall thickness compared to YC. SVF treatment significantly decreased wall thickness compared to OC (B). Main group effect P < 0.001, *P* < 0.05 vs. Young Control (*) and vs. Old Control (#). H&E data are analyzed with one-way ANOVA followed by Bonferroni post hoc test of following number of animals in each group: YC n = 4, OC n = 3, O+EC n = 3, O+SVF n = 7. Masson’s trichrome staining revealed an increase in % collagen content in OC compared to YC (C). Main group effect P = 0.010, *P* < 0.05 vs. Young Control (*). Trichrome data are analyzed with Kruskall-Wallis one-way ANOVA, YC: n = 3, OC n = 3, O+SVF n = 4. Data are presented as mean ± SD.

To corroborate LV size and thickness analyzed by ultrasound, histologic evaluation of a subset of hearts via hematoxylin and eosin (H&E) staining was performed. Microscopic examinations of H&E-stained cardiac sections under low magnification revealed significant thickening of the LV wall in all aged rats, regardless of treatment, compared to young control (**Fig 5A**). O+SVF hearts exhibited reduced LV thickness compared to OC (**Fig 5B**).

To assess potential changes in collagen content, a subset of hearts were sectioned and underwent Masson’s trichrome staining. There was a significant increase in collagen content in OC compared to YC (**Fig 5C**), and this was decreased following SVF treatment.

**Fig 6:**
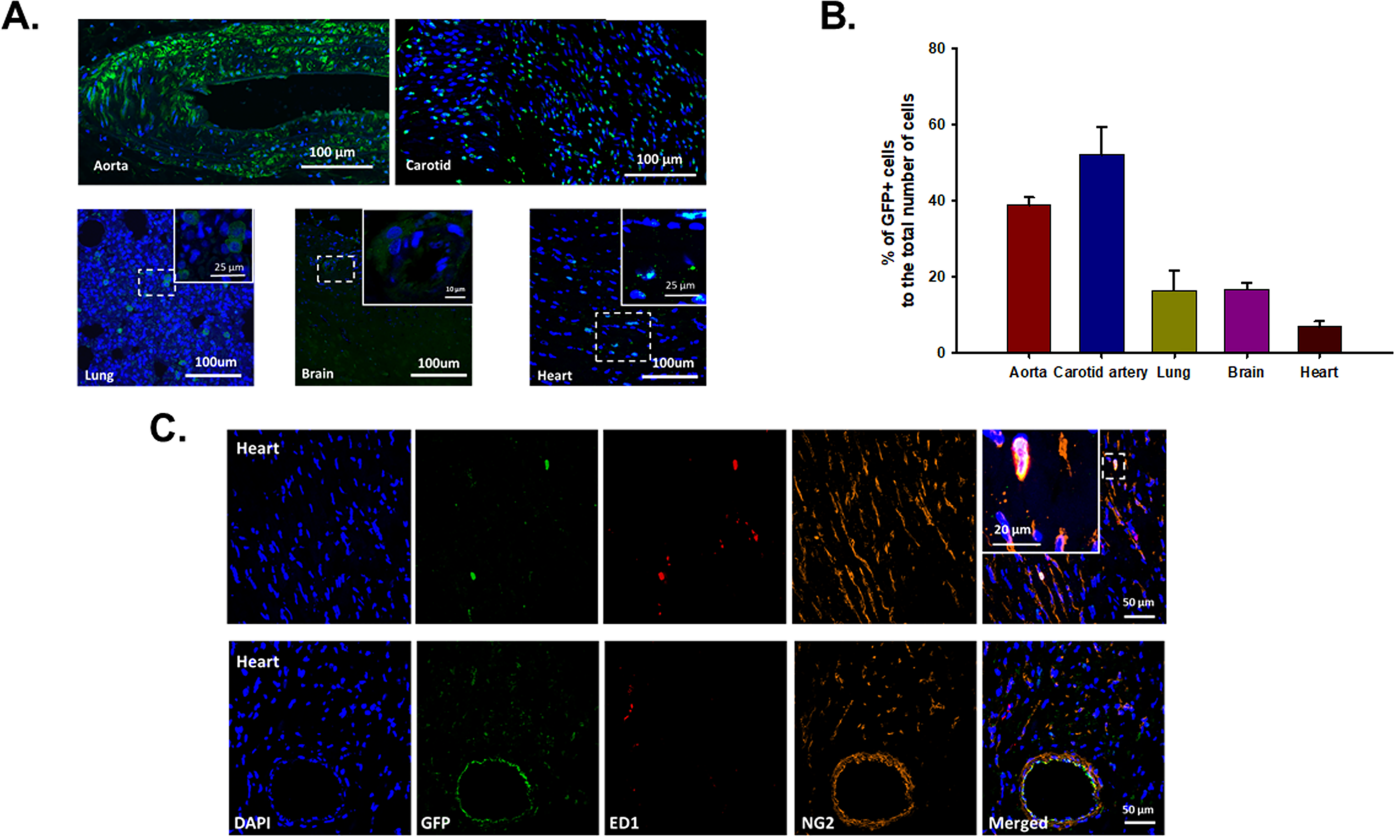
GFP positive cells in different organs four weeks following GFP^+^ SVF cell injection. GFP^+^ cells were found in aorta, carotid artery, lung, brain and heart, scale bar = 100μm (A). Graphical representation of percentage of GFP^+^ cells to total number of nucleated cells (DAPI, blue) in different organs (B). Co-localization of GFP (green), ED1 (macrophage marker, red), and NG2 (pericyte marker, orange) in heart, scale bar = 50μm and 20μm in the insert image (C). YC n = 4, OC n = 3, O+EC n = 3, O+SVF n = 7.

In order to identify injected GFP^+^ SVF cells, antigen retrieval using anti-GFP antibody was utilized in a subset of hearts, tissue and organs from the O+SVF group and compared to negative controls. Four weeks after SVF injection, IF staining showed the presence of incorporated GFP^+^ SVF cells in different organs and calculated as a percentage of nucleated cells (via DAPI staining); 7% of the nucleated cells within selected images from epi-, endo- and myocardium (minimum 5 images/heart) were GFP^+^ cells (**Fig 6A & 6B**).

To determine if treatment with SVF resulted in absolute changes to selected cell marker composition numbers or if incorporated SVF was co-localized to these markers, IF staining using NG2+ (pericyte marker), GS-1 (vascular cell marker), and ED-1 (macrophage marker) was performed. Within the heart, SVF cells were localized inside large coronary arteries (bottom row, **Fig 6C**), and they were positioned adjacent to NG2+ cells (**Fig 6C, Table 2**). Of GFP^+^ cells remaining in the heart upon explant, 41.41 ± 10.20% of them were also positive for NG2, while 40.26 ± 7.68% also stained positive for GS1 (**Table 2**). Phenotypic marker staining (GS1, NG2, ED1) upon explant between groups revealed no significant differences (**Table 2**).

**Table 2:** Immunohistochemistry staining in the heart for all groups. Cells were stained with ED1 (macrophage marker), NG2 (pericyte marker), and GS1 (vascular cell marker) and compared to total number (TN) of cells in images (assessed via DAPI). No significant differences in ED1, NG2 or GS1 were found between groups. O+SVF heart images were stained with anti-GFP, and co-stained with either ED1, NG2, and GS1. Most of the GFP^+^ cells found in the heart four weeks after injection were also positive for NG2 or GS1 rather than ED1. YC n = 4, OC n = 3, O+EC n = 3, O+SVF n = 7.

	YC	OC	O+EC	O+SVF
**ED1:TN**	5.64±0.23%	6.71±2.32%	4.32±0.82%	6.25±0.44%
**NG2:TN**	44.59±5.80%	48.3±4.21%	46.18±3.32%	40.77±2.75%
**GS1:TN**	54.48±3.47%	46.89±6.05%	54.21±4.20%	55.70±2.51%
**GFP:TN**				6.89±1.49%
**(GFP/ED1):GFP**				9.96±1.95%
**(GFP/NG2):GFP**				41.41±10.20%
**(GFP/GS1):GFP**				40.26±7.68%

### Telemetry

To evaluate arrhythmias caused by systemic delivery of SVF cells, indwelling biopotentials were captured in conscious animals weekly for four weeks in a subset of rats from OC and O+SVF groups. Electrocardiographic parameters were analyzed using Lab Chart EKG Analysis Module which graphed isoelectric noise and form factor from RR intervals and showed no arrhythmias in either OC or O+SVF rats following cell injection (data not shown). Abnormal readings that were caused by the rat’s movement were discarded from analysis.

## Discussion

The major findings from the present study are as follows: 1) GFP^+^ cells persisted in a variety of tissues in old female rats up to four weeks post-i.v. injection, 2) aged rats receiving SVF cell injection exhibit improved diastolic function compared to O+EC and OC groups, 3) O+SVF rats demonstrate increased CFR compared to baseline assessment, and 4) perfusion of LV was enhanced after 4 weeks of SVF injection.

Four weeks following a single i.v. injection of GFP^+^ SVF cells (6x10^6^/rat), GFP^+^ cells were largely populated in conducting artery walls as previously shown (**Fig 6B**) [22]. Our data shows that 7% of nucleated cells within the randomized regions of cardiac tissue were GFP^+^ in O+SVF hearts (**Fig 6B**), but whether the observed GFP^+^ cells incorporated into the arteries and organs directly or were generated via subsequent cell division is unclear [22]. Regardless, other methods of cell therapy delivery (direct intracoronary injection, for example) in humans have exhibited relatively poor retention and survivability immediately post-injection, demonstrating 1.3 to 2.6% remaining in ischemic LV 50–75 minutes later [34]. The current results suggest i.v. injection may be a superior method to retain cells [22] for at least four weeks post-injection in aged non-infarcted rat hearts. Further, the GFP^+^ cells in the heart were located primarily near or in close proximity to large coronary vessels (**Fig 6C**), and ~40% were co-labeled with GS-1 (a vascular cell marker) and/or NG2 (pericyte marker) (**Table 2**). It has been shown that longer retention of regenerative cells in the tissue correlates directly with improvements in cardiac remodeling and ejection fraction following myocardial infarction (MI) [35]. Thus, it seems plausible that increased incorporation of GFP^+^ cells in the coronary vascular and perivascular regions likely improves coronary vascular function in the current study.

In the present study, we measured CFR using Doppler variables and microsphere perfusion in order to examine the relationship between coronary flow and LV functional parameters during aging. Our results showed that Doppler-derived CFR and diastolic parameters (E/A, IVRT) were adversely affected in our aged model, while systolic function (EF) was preserved (**Figs 1 and 2, Table 1**). This study strengthens the association between CFR and myocardial diastolic function; CFR decrease in subjects without evidence of coronary artery stenosis may contribute to diastolic impairment and subsequent coronary microvascular flow reduction [36]. Recent studies have shown that diastolic dysfunction is often silently developed, and CFR is already considerably reduced in patients with heart failure with preserved ejection fraction (HFpEF), even though systolic function appears normal or systolic dysfunction is not advanced [5, 37].

We chose to use well-established invasive techniques to assess regional cardiac perfusion. Microspheres allow for quantitative measurement of myocardial perfusion down to secondary or tertiary coronary arterioles during resting and stress conditions, offering an absolute BF calculation. In patients with non-obstructive CAD, abnormal CFR is a predictor of poor outcome [38]. An alternative CFR calculation, percentage of BF above resting, has been used to determine the presence and severity of CMD in patients without obstructive CAD [37]. We found decreased perfusion in septal regions of OC and O+EC hearts compared to YC (**Fig 3A**). O+SVF hearts exhibited increased BF reserve in septal and LV regions compared to both OC and O+EC (**Fig 3A**), due to significant increases in absolute LV BF post-dobutamine (**Fig 3B**). In contrast to the ultrasound results, we observed an age-related increase in resting perfusion across the entire LV, but these OC rats were unable to increase BF in response to dobutamine (**Fig 3B**). This increase in myocardial BF at rest in aged humans and similar hyperemic BF between young and old has been reported previously in patients using 13N-ammonia and positron emission tomography [39]. Nonetheless, we believe some important conclusions from this data relate to 1) the relatively low percentage above resting BF achieved in OC and O+EC groups (**Fig 3A**), but also 2) the similarities of absolute LV BF during both rest and hyperemia between YC and O+SVF (**Fig 3B**).

Contrary to our hypothesis, isolated coronary arterioles did not exhibit improvements to vasoreactivity in O+SVF group (**Fig 4**). Similarly, peripheral mesenteric arteries did not show any changes in endothelium-dependent or -independent vasodilation following cell treatment (**S1 Fig**). Surprisingly, treatment with SVF or EC lowered sensitivity to bradykinin at the low doses in arterioles from the coronary, but maximum vasodilation between groups was unchanged (**Fig 4**). Our data contradicts a prior study where age-related hyper-vasoconstriction to endothelin in coronary arterioles was shown [40], but this could be due to different sources of young control rats (NIA in LeBlanc et al. vs Harlan in the present study). It is also possible that other measures of vascular function and signaling not assessed in the current study may be significantly altered in coronary arterioles after SVF treatment in aged rats. For example, our lab has previously shown an age-related increase in reactive oxygen species production (H_2_O_2_) in coronary arterioles that contributes to flow-mediated vasodilation [19, 41]. Future studies should pursue the relationship between vasoreactivity and the known anti-oxidant properties of SVF once incorporated in the tissue [12].

Another point of discussion regarding the lack of isolated vessel improvements and the enhanced coronary flow in the O+SVF group involves the specificity of dobutamine, a selective beta-one adrenergic receptor agonist. It is well described that both aging and failing hearts undergo beta-receptor desensitization [24, 42]. The improvement in dobutamine-stimulated BF in O+SVF hearts following SVF treatment in the present study may be attributable to altered beta-adrenergic signaling in the aged myocardium/microvasculature and/or increased sensitivity to beta-one adrenergic receptors. Additionally, with advancing age there is a proportional decline in LV diastolic “lusitropic” function [43], and in vivo administration of dobutamine has been shown to reverse the lusitropic dysfunction in aged mice possibly through alterations in phospholamban levels [44]. SVF treatment may enhance the lusitropic function in response to dobutamine in aged rats, which would be independent of isolated vessel function. More experiments are needed to further elucidate these potential mechanisms.

The relative invasiveness in obtaining adipose tissues vs. other sources of regenerative cells (bone marrow, for example) makes freshly isolated SVF a desirable option to treat patients with CAD. In general, the SVF contains a high percentage of stromal elements: hematopoietic stem/progenitor cells (c-Kit, VEGFR2, CD34) and endothelial cells (CD31), leukocytes (CD45), myeloid cells (CD13, CD11b), and pericyte lineages [23, 45]. Of course, use of SVF in the clinic demands future investigation into autologous SVF, i.e. old cells into old models. Our laboratory has previously shown age-related reduction in the neovascular potential of adipose SVF, specifically in CD47+ (82.63 ± 1.89% in young SVF vs 69.26 ± 2.82% in old) and VEGFR2+ (18.73 ± 3.38% in young vs 9.96 ± 1.14% in old) cells [23], which led us to choose young rats for SVF isolation for the present study. If cell culturing prior to injection was an option for future clinical studies, it has been previously shown that paracrine activity of adipose-derived cells from aged animals can be enhanced following exposure to hypoxia [46, 47]. Further, hypoxia preconditioning can restore angiogenic capacity of adipose-derived stromal cells from aged humans [48]. How much this may effect *in vivo* cardiac function is questionable, as treatment with young SVF did not stimulate an increase in coronary vessel density (GS-1^+^) compared to other groups (**Table 2**).

Significant limitations to the current study must be discussed. While ultrasound data indicate improvements in CFR and diastolic function in O+SVF rats compared to OC, hemodynamic measurements of diastolic function (like LV pressure fall data, LA volume, pulmonary pressures at systole and diastole, e´ velocity) would further support and confirm the diastolic functional data. Pressure-volume relationship curves would allow us to measure pressure at the end of diastole, giving us end-diastolic pressure-volume relation. Also, assessing mean arterial pressure changes in the present study would be important to examine as driving force of the heart could significantly affect coronary conductance [49]. Future studies must also address the likely possibility that a specific cell type in the SVF is contributing to the improvement in coronary function in aged rats in a non-angiogenic mechanism (i.e. paracrine). For example, previous data from our collaborations point to a CD11b+ macrophage population in the SVF that has been shown to elicit changes in vasomotor tone in peripheral vessels [22], but the relationship between CD11b+ macrophages and coronary vessels are relatively unknown.

### Conclusion

Systemically-delivered syngeneic SVF cells disseminated and incorporated throughout the aged rat model. SVF treatment elicited enhanced coronary microvascular perfusion with an increase in CFR, as well as improved diastolic function in an aged female population compared to OC and O+EC. Our findings offer a promising autologous cell therapy targeting the specific cardiac and microvascular challenges facing elderly women, albeit mechanistic underpinnings remain inconclusive.

## Supporting information

S1 Fig Isolated mesenteric arterioles vasoreactivity. Endothelial-dependent (ACh) and–independent (SNP) vasodilation was assessed in isolated mesenteric arteries. Mesenteric arteries from YC rats could not maintain phenylephrine pre-constriction in order to test endothelial dependent/independent vasodilatation. No significant differences were observed between old groups’ pre-constriction levels or in either vasodilator response. Data are presented as mean ± SEM, analyzed with repeated measures ANOVA followed by Bonferroni post hoc test of following number of animals in each group: ACh, n = 9 for OC, n = 5 for Old+EC, n = 7 for Old+SVF; for SNP, n = 7 for OC, n = 5 for Old+EC, n = 7 for Old+SVF.
(TIF)

## Abbreviations

BF: Blood flow
BW: Body weight
CO: Cardiac output
CAD: Coronary artery disease
CFR: Coronary flow reserve
CMD: Coronary microvascular dysfunction
EF: Ejection fraction
ECG: Electrocardiogram
GFP: Green Fluorescent Protein
HW: Heart weight
i.v.: Intravenous
IVS;d: Interventricular septal end diastole
IVS;s: Interventricular septal end systole
LAD: Left anterior descending
LVDD: Left ventricular diastolic dysfunction
LVID: LV internal diameter
LVPW;d: LV posterior wall end diastole
LVPW;s: LV posterior wall end systole
PWT: LV posterior wall thickness
O+SVF: Old + GFP^+^ SVF cells
O+EC: Old + rat aortic endothelial cells
OC: Old control
RAEC: Rat aortic endothelial cells
SV: Stroke volume
SVF: Stromal vascular fraction
YC: Young control
