# Supplementary figures and images for: Adipose-derived cells improve left ventricular diastolic function and increase microvascular perfusion in advanced age
Source: PLoS One. 2018 Aug 24;13(8):e0202934. doi: 10.1371/journal.pone.0202934 (PMC6108481; doi:10.1371/journal.pone.0202934)

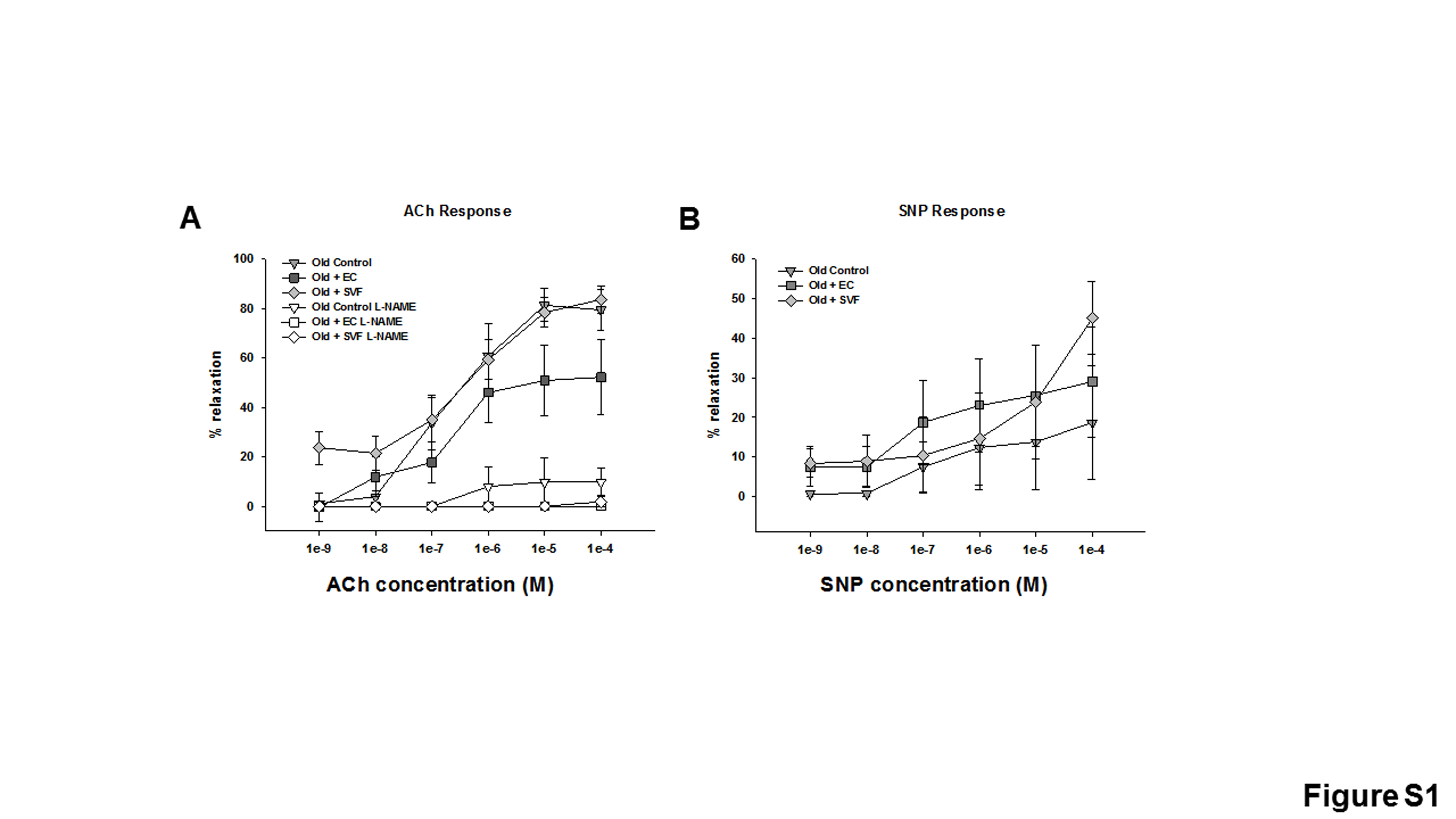

Supplement: S1 Fig — Endothelial-dependent (ACh) and–independent (SNP) vasodilation was assessed in isolated mesenteric arteries. Mesenteric arteries from YC rats could not maintain phenylephrine pre-constriction in order to test endothelial dependent/independent vasodilatation. No significant differences were observed between old groups’ pre-constriction levels or in either vasodilator response. Data are presented as mean ± SEM, analyzed with repeated measures ANOVA followed by Bonferroni post hoc test of following number of animals in each group: ACh, n = 9 for OC, n = 5 for Old+EC, n = 7 for Old+SVF; for SNP, n = 7 for OC, n = 5 for Old+EC, n = 7 for Old+SVF. (TIF) [file pone.0202934.s001.tif]
